# Efficient-ECGNet framework for COVID-19 classification and correlation prediction with the cardio disease through electrocardiogram medical imaging

**DOI:** 10.3389/fmed.2022.1005920

**Published:** 2022-11-04

**Authors:** Marriam Nawaz, Tahira Nazir, Ali Javed, Khalid Mahmood Malik, Abdul Khader Jilani Saudagar, Muhammad Badruddin Khan, Mozaherul Hoque Abul Hasanat, Abdullah AlTameem, Mohammed AlKhathami

**Affiliations:** ^1^Department of Computer Science, University of Engineering and Technology, Taxila, Pakistan; ^2^Department of Software Engineering, University of Engineering and Technology, Taxila, Pakistan; ^3^Department of Computer Science, Faculty of Computing, Riphah International University Gulberg Green Campus, Islamabad, Pakistan; ^4^Department of Computer Science and Engineering, Oakland University, Rochester, NY, United States; ^5^Information Systems Department, College of Computer and Information Sciences, Imam Mohammad Ibn Saud Islamic University (IMSIU), Riyadh, Saudi Arabia

**Keywords:** computer vision, COVID-19, deep learning, ECG, Efficient-ECGNet, medical imaging

## Abstract

In the last 2 years, we have witnessed multiple waves of coronavirus that affected millions of people around the globe. The proper cure for COVID-19 has not been diagnosed as vaccinated people also got infected with this disease. Precise and timely detection of COVID-19 can save human lives and protect them from complicated treatment procedures. Researchers have employed several medical imaging modalities like CT-Scan and X-ray for COVID-19 detection, however, little concentration is invested in the ECG imaging analysis. ECGs are quickly available image modality in comparison to CT-Scan and X-ray, therefore, we use them for diagnosing COVID-19. Efficient and effective detection of COVID-19 from the ECG signal is a complex and time-taking task, as researchers usually convert them into numeric values before applying any method which ultimately increases the computational burden. In this work, we tried to overcome these challenges by directly employing the ECG images in a deep-learning (DL)-based approach. More specifically, we introduce an Efficient-ECGNet method that presents an improved version of the EfficientNetV2-B4 model with additional dense layers and is capable of accurately classifying the ECG images into healthy, COVID-19, myocardial infarction (MI), abnormal heartbeats (AHB), and patients with Previous History of Myocardial Infarction (PMI) classes. Moreover, we introduce a module to measure the similarity of COVID-19-affected ECG images with the rest of the diseases. To the best of our knowledge, this is the first effort to approximate the correlation of COVID-19 patients with those having any previous or current history of cardio or respiratory disease. Further, we generate the heatmaps to demonstrate the accurate key-points computation ability of our method. We have performed extensive experimentation on a publicly available dataset to show the robustness of the proposed approach and confirmed that the Efficient-ECGNet framework is reliable to classify the ECG-based COVID-19 samples.

## Introduction

Coronavirus Disease 2019 (COVID-19) has quickly spread through enhanced mortalities across the globe ahead of a long-lasting worldwide pandemic. Initially, this virus came out from China in December 2019, and since then has caused approx. 2 million deaths worldwide. According to the report: approx. 5.2 million fatalities have been recognized globally and over 261 million instances have been documented as of November 2021 (1). The severe acute respiratory syndrome coronavirus 2 (SARS-CoV-2) disease frequently disturbs the respiratory system with a great potential to cause the failure of multiple organs. Furthermore, this pandemic virus connects to an angiotensin-converting enzyme (ACE2) that appears in the heart, kidneys, lungs, etc. These types of failures have a proliferative effect on the cardiovascular system (2–4). The evolution in biomedical applications through artificial intelligence (AI) has facilitated in creation of accomplished models for consistent computer-aided diagnostic (CAD) conclusions. Such systems tried to overcome the burden of the healthcare accommodations like medical experts (5, 6).

The abrupt pandemic namely COVID-19 is spreading at high speed across the globe and has urged the research community to present techniques that can recognize it at its earliest state. Several image modalities like X-ray, CT-Scan, and ECG signal-based images are used for this purpose (7). Extensive work for COVID-19 detection and classification is concerned with CT-scans, and chest X-rays-based image modalities (8–10). However, there are limited methods which are employing ECG signals for the detection and recognition of COVID-19. The focus of this work is on the employment of ECG images for the detection and classification of the COVID-19 virus. An ECG is the primary diagnostic instrument and non-invasive recordings which are used to translate the ECG signals. Moreover, ECG measures the activities and actions of the heart from various directions, i.e., 12 leads. The ECG images are further divided into numerous categories, i.e., myocardial infarction (MI), abnormal heartbeats (AHB), Patients with Previous Histories of Myocardial Infarction (PMI), Normal ECG, and ECG of the COVID-19 affected patients. Employment of ECG signal-based images for the COVID-19 detection and classification is grasping the attention of researchers due to their economic feasibility, and safety. Moreover, the real-time monitoring of ECG signals can help the practitioners to quickly locate the affected regions. However, ECG images need experts to examine them which can delay the detection process (11, 12). Moreover, existing ECG-based COVID-19 recognition frameworks are concerned with converting the input samples into statistical data and performing the required computation, which in turn causes an increase in the computational cost and the detection time. Therefore, there is a need for such an automated system that can directly read the ECG images and recognize COVID-19 patients.

Accurate recognition and automatic classification of COVID-19 is still challenging work due to the varying nature of this virus. Furthermore, variations in ECG images like size, location, QRS values, peaks of ECG, and 12 leads values also complicate the recognition procedure of COVID-19. Moreover, the samples having various cardiac diseases like MI, AHB, etc., can also degrade the detection accuracy of existing methods. So, there is also a lack of such a model which can identify the coronavirus victims directly from ECG images and identify the similarity between COVID and other cardiac diseases. Consequently, there is a demand to propose a COVID-19 recognition system that can timely detect and differentiate COVID-19 victims from other cardiac patients *via* using ECG images and measuring the similarity between them. To cope with the above-mentioned challenges, we have presented a novel framework namely Efficient-ECGNet with additional added dense layers which are not only capable of accurately diagnosing the COVID-19 samples but can also classify among the other heart abnormalities. Additionally, we have introduced a module to find the similarity among the mentioned diseases. The details of the entire framework are discussed in the subsequent sections. The presented method can automatically recognize COVID-19 from ECG images without converting the signal into statistical values and is robust to COVID-19 recognition. The main contributions of our work are given below:

• We propose a novel Efficient-ECGNet method for effective deep features computation to enhance the recognition and classification performance of ECG images into five classes, i.e., healthy, COVID-19, MI, AHB, and patients with PMI.
• The method is introduced to find the correlation of COVID-19 with other cardio diseases to better understand the nature of this deadly virus.
• We present a computationally efficient framework for coronavirus classification because of minimum network parameters.
• We generate the heatmaps to show the accurate key-points computation ability of the proposed model.
• Rigorous comparison is accomplished with state-of-the-art frameworks to verify the worth of the presented technique.

The rest of our work is planned as follows. Section “Related work” covers the work already performed by the researchers for the COVID19 classification, while the details of the introduced work are defined in section “Materials and methods”. We have discussed the results of the proposed method in section “Results and discussion,” while in section “Conclusion” we draw the conclusion of the proposed work.

## Related work

In this part, a detailed analysis of existing approaches used for the detection and classification of COVID-19 samples is performed. For this reason, several image modalities for example X-rays, CT-Scan and ECG signal-based images have been heavily explored by the research community. We have investigated the existing approaches along with their critical analysis.

Initially, most of the research work was focused on using chest X-rays and CT-Scan images to perform the COVID-19 detection and classification task. Arora et al. (13) utilized the lung CT-Scan samples to locate the COVID-19-affected images. To accomplish this task, the work in Arora et al. (13) initially employed the residual dense neural network (RDNN) to enhance the visual representation of suspected images. Initially, the image preprocessing step is performed, and then the diverseness of input data is improved *via* using a data augmentation step. In the last stage, numerous DL techniques like DenseNet, VGG-16, Inception-V3, MobileNet, XceptionNet, and residual network were applied to classify the normal and coronavirus samples. The model presented in Arora et al. (13) exhibits better COVID-19 detection, however, the approach is suffering from high computational cost. In Panwar et al. (14), a DL method was presented for the automatic identification and recognition of coronavirus samples *via* employing two types of image modalities namely the CT-Scan and X-ray images. The author proposed an improved version of the VGG-19 approach by presenting five additional layers in the original framework to extract a set of deep image key-points and perform the classification task. This work (14) is robust to COVID-19-affected images, however, the classification accuracy requires more enhancement. Rahimzadeh et al. (15) presented a model to identify and classify the COVID-19 samples by using the Feature pyramid network (FPN) along with the DL-based approach namely ResNet50V2. This technique (15) performs well for COVID-19 image classification, however, does not generalize well for real-world scenarios. Another work was presented in Turkoglu (16) to automatically identify and categorize the coronavirus images *via* employing the chest CT-Scan samples. After accomplishing a preprocessing phase, a method named DenseNet201 was applied to calculate the feature vector of the input samples. In the next step, the computed feature vector was used to train the Extreme Learning Machine (ELM) (17) approach to execute the categorization job. In the last step, a voting procedure was employed to decide the final classification results. This approach (16) is effective for COVID-19 image recognition and classification tasks, however, the model needs huge data for network training which in turn enhances the computational burden.

Rahimzadeh and Attar (18) proposed an approach to categorize coronavirus and pneumonia victims by using chest X-ray modalities. The work was performed by merging the features computed from two frameworks namely the Xception and residual network, and then the classification task was accomplished over the deep features *via* using the CNN classifier. The method presented in Rahimzadeh and Attar (18) works well for the COVID-19-affected samples, however, suffering from a high computing cost. Another work was presented in Ahuja et al. (19) to identify and categorize the COVID-19 images with the employment of CT-Scan samples. In the first step, a data augmentation approach was used to enhance the size and variety of training data. After performing the preprocessing step, numerous DL-based methods like three variants of the residual net with 18, 101, and 50 CNN layers were used for deep features computation. Furthermore, the SqueezeNet was also used to locate and classify the coronavirus and pneumonia-affected images. This work (19) exhibits enhanced COVID-19 classification performance, however, the proposed solution is evaluated on a small dataset. Furthermore, in Garain et al. (20) a lightweight model comprising three CNN layers was presented to recognize and categorize the coronavirus by utilizing the CT-Scan image modality. In the first step, a preprocessing step employing the Gabor filter was used to improve the visual representation of the input images. Then, an intensity-to-latency encoding approach was used to generate a wave of spikes which was passed to numerous convolved and pooling layers and reached the penultimate layer. In the final step, the extracted feature vector was used to perform the sample categorization task. The work presented in Garain et al. (20) is robust to the coronavirus classification task, however, requires extensive model training data. Jain et al. (21) presented a method to locate and classify the coronavirus-affected images with the help of chest X-ray modalities. Initially, a data augmentation step was performed over the training data to increase its diversity. In the next step, three different deep learning techniques named ResNet, Inception V3, and XceptionNet were used for features computation which was later used for COVID-19 classification. It is concluded in Jain et al. (21) that the XceptionNet approach outperforms the other techniques, however, it is unable to perform well for unseen images.

Kadry et al. (22) introduced an automated framework for the identification and categorization of COVID-19 samples. Initially, a preprocessing step utilizing the Chaotic-Bat-Algorithm and Kapur’s entropy (CBA+KE) algorithm was used to enhance the visual appearance of the suspected images. Secondly, the ROIs were identified from the input images by using the bi-level threshold filtering approach. After obtaining the ROIs, numerous methods combining the Discrete Wavelet Transform (DWT), Gray-Level Co-Occurrence Matrix (GLCM), and Hu Moments (HuM) approaches were used to compute the sample key points. Based on the computed features, several ML-based classifiers namely KNN, SVM, Random Forest (RF), and Naive Bayes (NB) were employed to execute the categorization task. This work (22) obtained the highest results with the SVM classifier, however, the classification results need further enhancements. Another work employing both the CT-Scan and X-ray image modalities was presented in Mukherjee et al. (23). A 9-layered CNN framework was presented in Mukherjee et al. (23) to detect and classify the healthy and COVID-19-affected images. This work (23) presents a low-cost solution to the COVID-19 classification task, however, the sample categorization performance needs further improvements. In Sharma (24), a residual network-based approach was introduced to locate the COVID-19 images which showed better accuracy for the coronavirus classification task, However, this method (24) required huge processing power. Herath et al. (25) proposed a CNN-based approach to identify the coronavirus *via* using the lung’s CT-scan samples. The technique presented in Herath et al. (25) exhibits improved virus categorization performance, however, evaluation needs further analysis on a large and complicated database. Moreover, an approach utilizing both the X-ray and CT-Scan images was introduced in Sedik et al. (26). The classification framework employed a five-layered CNN network together with the short-term memory (LSTM) approach to locate and categorize the COVID-19 patient samples. This work demonstrates improved coronavirus identification performance, however, needs huge samples to perform the network training process.

Several techniques employed the ECG signal-based images to detect and classify the COVID-19-affected images due to their economic feasibility, easier availability, safety, and real-time monitoring. An ECG image-based COVID-19 classification framework was presented by Rahman et al. (4). After performing the preprocessing step, six DL-based methods namely MobileNetv2, ResNet-18, 50, 101, DenseNet-201, and Inception-V3 were employed to compute the deep features from the input samples and execute the categorization task. This work (4) categorized the suspected samples into five classes namely healthy, coronavirus affected, MI, AHB, and recovered myocardial infarction (RMI). The approach in Rahman et al. (4) attains the highest accuracy for the DenseNet-201, however, suffering from high computational cost. Another work was proposed in Ozdemir et al. (27) by employing a CNN-based classifier. Initially, the hexaxial feature mapping was proposed to transform the ECG signal into colorful samples. Then, the GLCM approach was applied to calculate the features from the input image. In the last step, the computed image key-points were used to train the CNN classifier to categorize the healthy and coronavirus samples. The work (27) is robust to COVID-19-affected images, however, performance needs further enhancements. Similarly, another work was presented in Angeli et al. (1) to recognize the COVID-19 affected victims by using the ECG image, however, needs further evaluations to better explain their results. Rahman and Hossain (28) also employed the ECG images for the coronavirus classification. Initially, the ECG signal was transformed into a 2D image spectrogram. Then a data augmentation phase was used to enhance the dataset size and its diversity. In the next step, the images were passed to a CNN framework comprising seven convolution layers for feature computation and categorization. The method (28) shows better COVID-19 classification performance, however, may not generalize well to real-world scenarios.

From the above-discussed techniques, it can be concluded that even though numerous techniques have been presented by the research community for the early diagnosis and classification of COVID-19-affected samples. However, there is room for performance improvements both in terms of COVID detection and model computational complexity. Moreover, it can be seen that little work is presented for COVID-19 detection using the ECG signal-based images without focusing on the explainability aspect of the DL method. So, there is an extensive gap to present more robust techniques for COVID-19 detection and classification *via* employing the ECG images due to their safe, reliable, and cost-effective nature.

## Materials and methods

**FIGURE 1:**
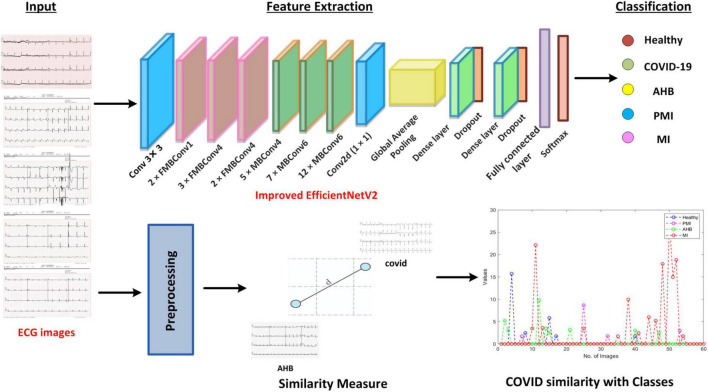
Workflow diagram of ECG image-based COVID-19 classification using Efficient-ECGNet.

In the presented work, a novel framework namely Efficient-ECGNet is introduced to detect and classify the suspected samples into five possible classes named healthy, COVID-19, PMI, MI, and AHB, respectively. Furthermore, we have introduced a module to analyze the COVID-19-affected samples with other heart diseases to show their similarity. Descriptively, we have modified the EfficientNetV2-B4 framework by adding dense layers at the end of the architecture to better analyze the input images. The technique comprises the following steps: (i) initially, we prepared the data and model by dividing the dataset into train and test sets and adjusted the hyper-parameters of network architecture, (ii) we used the EfficientNetV2 with the B4 base network with additional added dense layers for deep features computation and classification task, (iii) Finally, we have found the similarity of COVID-19 samples with other diseases to show its relevance with other diseases. The entire flow of the introduced technique is shown in Figure 1.

### Dataset

**FIGURE 2:**
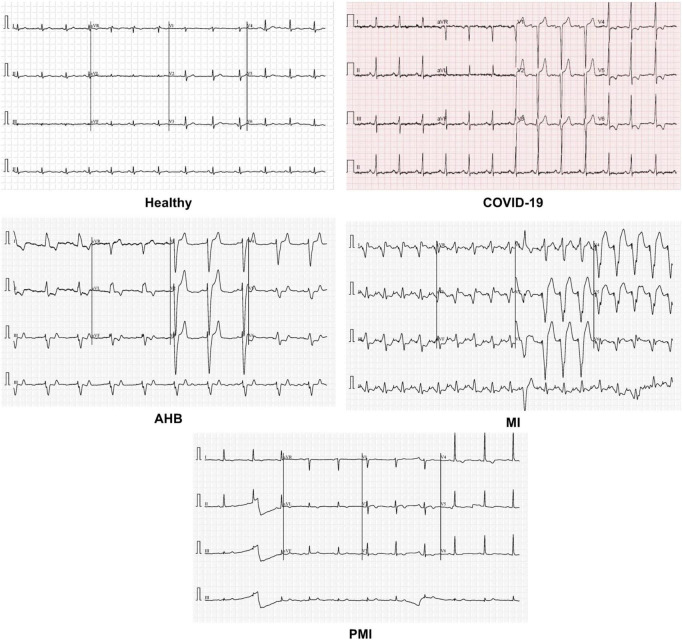
Sample images of all classes.

To check the COVID-19 identification and categorization performance of our method, a standard publicly available dataset (29) containing the ECG samples from healthy, COVID-19-effected, and cardiac patients is utilized (as shown in Figure 2). The dataset used 12-lead-based standard ECG images collected from EDAN SERIES—3 devices of 500 Hz sampling rate to align the four channels of a one-dimension signal. All ECG images have been annotated by several medical experts. This database comprises a total of 1,937 images from five different categories namely Healthy, AHB, MI, PMI, and COVID-19. The MI category contains the samples of patients with a critical coronary disease that happened because of obstruction of blood flow to different areas of the heart which ultimately results in a heart attack. The AHB category comprises the ECG images for those victims who are suffering from breath shortness or respirational issues after recovering from other cardiac diseases. While the PMI class consists of samples of the patients who remained a victim of MI in the past. Whereas the COVID class contains ECG images of people suffering from the COVID-19 pandemic. And the last category namely normal comprises images of healthy persons. There is a total of 77, 548, 203, 250, and 859 images from the MI, AHB, PMI, COVID, and normal classes, respectively.

### Efficient-ECGNet

To attain better classification results, it is important to achieve a robust set of image features because they contribute directly to differentiating the various categories of input samples. The employment of dense DL networks can assist to compute a more efficient set of features, which in turn causes to increase recall rate of approaches as well (30). At the same time, the usage of deep networks puts the computational constraint on the models as the deployment of these CNN approaches is highly dependent on the accessibility of processing power and memory requirements. Therefore, there is always a tradeoff between evaluation results and computational cost. Thus, there is an urgent need of introducing such a framework for COVID-19 recognition that can show better classification accuracy and maintain the computational cost as well (31). In this work, we introduced a lightweight and computationally robust model to enhance the model performance for classifying several abnormalities namely healthy, COVID-19, PMI, MI, and AHB-affected ECG samples, respectively.

The presented Efficient-ECGNet framework introduced an improved EfficientNetV2-B4 model. The EfficientNetV2 is an extended form of the EfficientNet (32). The aim of presenting the modified EfficientNetV2 model is to boost available resources while preserving the robust recall rate as well. The modified EfficientNetV2 model is designed by employing a light and proficient composite scaling approach that permits a standard ConvNet to be resized to any resource constraints while reserving the framework competence. Hence, the presented framework provides an optimal solution to both computational cost and optimum selection of network structure, i.e., the number of network layers or size of the sample feature vector. The EfficientNetV2 approach comprises a small number of model parameters and executes the classification task robustly. Moreover, compared to other latest DL models like AlexNet (33), GoogleNet (30), ResNet (34), and DenseNet (35), MobileNet (36), the EfficientNet approach attained efficient results both in the forms of categorization results and time complexity.

**FIGURE 3:**
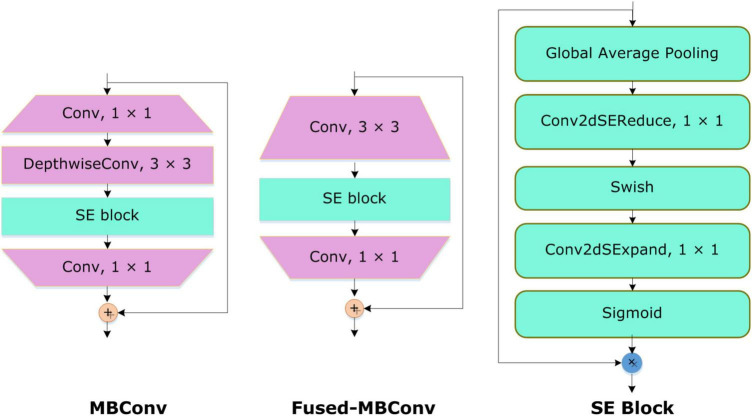
Visual demonstration of MBConv4, Fused-MBConv4, and SE blocks.

The inspiration for applying the EfficientNetV2 with additive dense layers for classifying the COVID-19-affected ECG-based images is that this approach is a lightweight framework with small inference and training time, and comprises few model parameters. To boost the classification accuracy and minimize the feature vector size and training time, the EfficientNetV2 approach utilizes the neural architecture search (NAS). Furthermore, the inclusion of the Fused-MBConv (FMBConv) blocks (37) in the EfficientNetV2 model optimizes the operative power and effectively uses mobile or server accelerators. While in comparison, just MBConv blocks (38) are employed as the main building block in the traditional EfficientNet approach which contains depth-wise convolutions only. Even though, the depth-wise convolutions minimized the number of arithmetic operations being performed, however, they lack to completely use new hardware accelerators. To obtain the computational advantage, the EfficientNetV2 approach comprehensively employs both MBConv and FMBConv blocks. In the FMBConv, the regular 3 × 3 convolution layers are introduced to replace the depth-wise 3 × 3 convolution and expansion 1 × 1 convolution of MBConv (Figure 3). The main objective of replacing certain MBConv with the FMBConv is to increase the execution speed of the framework while maintaining the categorization results (39).

To accomplish the COVID19 categorization task, the EfficientNetV2 model with the B4 base network is utilized. The main reason to choose the B4 base is that it demonstrates a reasonable trade-off between the model classification performance and execution time. An in-depth demonstration of the improved EfficientNetV2 model is mentioned in Table 1. The FMBConv blocks are used at the starting layers of the improved EfficientNet-V2 model while at the advanced level, the MBConv blocks with 3 × 3 and 5 × 5 depth-wise convolutions together with squeeze-and-excitation block (SEB) (40), and swish activation is used. To obtain the robust classification results, the employed MBConv blocks maintain an inverted residual connection with the SEB. Furthermore, the SEBs use the attention mechanism to improve key-points representations which enables the network to rank affected areas of ECG image *via* employing the self-learning weights. A detailed structural description of SEBs can be found in Figure 3. The framework employs the swish activation function (SAF) (41) as an alternative to ReLU as the ReLU activation function (ReLUAF) eliminates the values lower than zero and causes to loss of the important aspect of the employed ECG signal. The SAF can be computed by using Equation (1).


(1) SAF(x)=x×sigmoid(x)

**TABLE 1:** Architectural details of the proposed framework.

Block/Layer	Size	Channel
BatchNorm	224×224	3
ConvL (3 × 3)	112 × 112	40
2 × FMBConv1	112 × 112	16
3 × FMBConv4	56 × 56	40
2 × FMBConv4	28 × 28	56
5 × MB_Conv4	14 × 14	112
7 × MB_Conv6	14 × 14	136
12 × MB_Conv6	7 × 7	232
Conv2d (1 × 1)	7 × 7	232
GlobalAveragePooling layer	1,792	
Denselayer	128	
Dropout	128	
Denselayer	64	
Dropout	64	
FC		
Classification		

Furthermore, to down-sample the input image sizes, a Batch normalization layer is introduced at the beginning of a framework. We used only 3 FMBConv blocks as they comprise extensive parameters for large values of *O* (total output channels) (Table 1). Moreover, to avoid the problem of model over-fitting, a global average pooling layer is also added after the MBConv blocks to minimize the model parameters. We further introduced 2 additional inner dense layers accompanying the ReLUAF and dropout layers which assist in extracting the more efficient set of sample key-points by presenting them in a viable manner. To boost the model performance, a dropout rate of 30% is selected randomly. At last, a dense layer comprising five output neurons along with the Softmax activation method is used to design a fully automated COVID-19 classification model.

### Loss function

Models employ the loss function (LF) to check their effectiveness. For large-sized data, the networks utilize automatic learning to detect the rules and give estimations. The main objective of the LF is to compute the extent of change in the real and estimated scores. The LF is frequently updated in the model training procedure till the robust fitting value is acquired to minimize the error. For classification tasks, the cross-entropy LF employs the Softmax function to validate the variance among the computed and original values. The cross-entropy LF is calculated as:


(2) $$L=\frac{1}{N}\,\sum_{k=1}^{N}\log\left(\frac{e^{s_{j}}}{\sum_{i}e^{s_{k}}}\right)$$

Here, *N* is showing the total neurons in the final layer while*s_k_* is depicting the input vector.

### Similarity of COVID-19 samples with other diseases

After performing the classification task, here, we executed another estimation to compute the similarity of coronavirus-affected ECG signal with other abnormalities. *Furthermore, it is vital to discuss that according to the best of our knowledge, this is the first effort to approximate the similarity of COVID lesions with other classes, i.e., healthy, MIP, MI, and AHB samples, respectively*. To accomplish this, we take ECG images of all the classes and then prepare them to calculate the similarity between different images of all classes. For estimating the similarity of COVID samples with other images, different preprocessing operations are necessary. For this purpose, firstly we crop the images to remove the extra information. Secondly, the background of images is removed through a thresholding process (42) by using Equation 3.


(3) $$\begin{array}{l} Img_{b}^{j}(x,y)=\\ \{\begin{array}{c} 0\;if(\mu_{j}-\rho *\sigma_{j})\leq Img_{avg}^{j}(x,y)\leq (\mu_{j}+\rho *\sigma_{j})\\ 1\;\;\;\;\;\;\;\;\;\;\;\;\;\;\;\;\;\;\;\;\;\;otherwise \end{array} \end{array}$$

Here, $Img_{b}^{j}(x,y$) is showing the binary image, while $I\,m\,g_{a\,v\,g}^{j}\,\left(x,y\right)$ is presenting the average image. Moreover, μ_*j*_, and σ_*j*_ are showing the mean and variance for $I\,m\,g_{a\,v\,g}^{j}\,\left(x,y\right)$, and ρ is a positive real constant. After that, we performed the morphological operations, i.e., erosion and dilation to get the desired ECG signals by using Equation 4.


(4) $$I\,m\,g_{m\,o\,r}^{j}\,\left(x,y\right)=I\,m\,g_{b}^{j}\,\left(x,y\right)\,⊗\text{SE}$$

Here, $I\,m\,g_{m\,o\,r}^{j}\,\left(x,y\right)$ is showing the obtained image, while *SE* is a structuring element having disk-shaped and ⊗ is presenting the morphological operator. Then to measure the similarity of COVID-19-affected ECG images with the rest of the classes, we computed the Euclidean Distance (43) among them by using Equation 5.


(5) $$D\,i\,s_{u}^{2}\,\left(i\,m\,g_{i},i\,m\,g_{j}\right)=\sum_{n=1}^{M\,N}{\left(i\,m\,g_{i}^{n}-i\,m\,g_{j}^{n}\right)}^{2}$$

Here, *img*_*i*_ and *img*_*j*_ are showing the two images, while *MN* is presenting the resolution of the respective images and *n* is total samples. Based on the estimation, we measured the class which is more related to the COVID-19 sample. The entire method is explained in Algorithm 1.

**Algorithm 1:** Phases to measure the COVID similarity with other abnormalities

**START** **INPUT:** ECG_Samples **OUTPUT:** Similarity score **ECG_Samples:** Images from classes **I(x)= COVID samples** **Similarity score:** estimation of COVID similarity with other four classes **ECG_samples** ← [x,y] **// Preprocessing step** **Crop_ECG_samples** ← Crop (ECG_samples) **Processed_ECG_samples** ← BackGroudRemoval (Crop_ECG_samples) **Enhanced_ ECG_samples** ← Morphological Operation(Processed_ECG_samples) **Resized_ECG_samples** ← **i**mresize(Enhanced _ECG_samples**)** **// Similarity Estimation** **Dataset** ← Path of Resized_ECG_samples from saved directory **DestinationFolder** ← Path to save the results **filePattern** ← fullfile(Dataset, ‘*.jpg’); **jpegFiles** ← dir(filePattern); **for** k = 1: length(jpegFiles) **baseFileName** ← jpegFiles(k).name; **fullFileName** ← fullfile(Dataset, baseFileName); **ECG_sample[J]** ← imread(fullFileName); **Estimated_Similarity(k)** ← sqrt(sum((I(:) - ECG_sample(:)) .∧ 2)); **End** SaveResult() and output Similarity score **FINISH**

## Results and discussion

In this section, we have provided an in-depth description of the utilized performance metrics. Furthermore, a comprehensive demonstration of obtained results for the employed model together with the comparison of other approaches is also discussed.

### Implementation details

We have implemented the introduced method in Python language with Keras TensorFlow libraries. The proposed approach is trained on the ECG samples by employing the stochastic gradient descent (SGD) optimizer. Furthermore, the early stopping regularization approach is utilized during model training to prohibit the problem of overfitting. We have trained the proposed approach for 20 epochs as we observed no significant change in our model’s performance after 20 epochs. Additional training parameters include the batch size and learning rate which are 12 and 0.0001 for our work. The detailed description of model parameters is discussed in Table 2. We have implemented the proposed approach on an Nvidia GTX1070 GPU-based system with 16GB RAM.

**TABLE 2:** Details of network hyperparameters.

Parameters	Value
Total used epochs	20
Used value of learning rate	0.0001
Selected batch size	12
Optimizer	SGD

**FIGURE 4:**
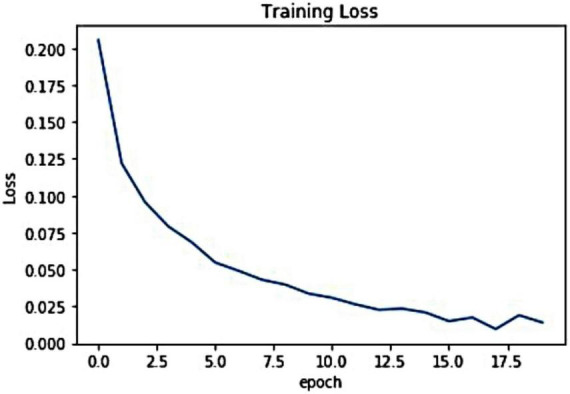
Pictorial demonstration of training loss.

**FIGURE 5:**
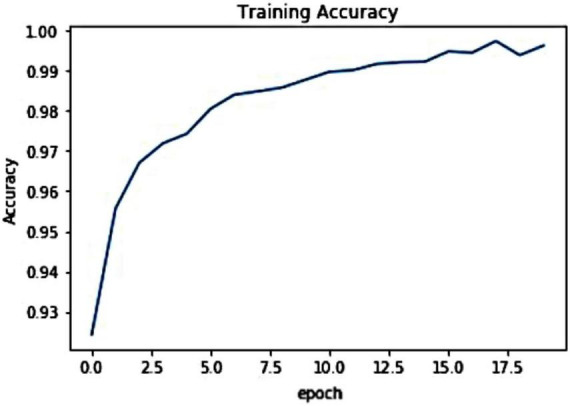
Training accuracy curve.

For the introduced framework, we have exhibited the pictorial depiction of the optimal loss graph in Figure 4. One can see from Figure 4 that our work reached the optimal score of 0.0023 at the 20th epoch, which is clearly elaborating the robust training of the presented model. Besides, we have shown the training accuracy curve in Figure 5 and acquired the value of 99.01%.

### Evaluation metrics

Numerous standard evaluation parameters, i.e., accuracy, precision, recall, and F1-score are employed to validate the COVID-19 recognition power of the proposed approach. The accuracy metric is computed as:


(6) $$A\,c\,c\,u\,r\,a\,c\,y=\frac{T\,r\,u\,e_{+}+T\,r\,u\,e_{-}}{T\,r\,u\,e_{+}+F\,a\,l\,s\,e_{+}+T\,r\,u\,e_{-}+F\,a\,l\,s\,e_{-}}$$

While the precision, recall, and F1- measure are calculated by using Equations (7)–(9), respectively.


(7) $$\text{Precision}=\frac{T\,r\,u\,e_{+}}{T\,r\,u\,e_{+}+F\,a\,l\,s\,e_{+}}$$

(8) $$\text{Recall}=\frac{T\,r\,u\,e_{+}}{T\,r\,u\,e_{+}+F\,a\,l\,s\,e_{-}}$$

(9) $$\mathrm{F1}\,\_\,\mathrm{score}=2\times \frac{\text{Precision}\times \text{Recall}}{\text{Precision}+\text{Recall}}$$

### Performance evaluation

In this part, we have accomplished three types of tests to show the efficacy of the proposed work for the COVID-19 classification from the ECG images. In the first analysis, we have demonstrated the class-wise results of the proposed solution, while in the second analysis, we have presented the Explainability analysis to illustrate the accurate key-points computation capability of the proposed method. Finally, we have shown the similarity of the ECG images of the COVID-19-effected patients with other types of ECG signals, i.e., ECG images of normal and cardiac-affected patients.

#### Classification results

To have an effective COVID-19 identification and categorization framework, it should be competent of distinguishing the COVID-19-effected ECG images from other types of samples, i.e., MI, PMI, normal, and AHB patient samples. To validate this scenario, we have performed an analysis by using the images of all categories from the dataset given in Khan et al. (29).

**FIGURE 6:**
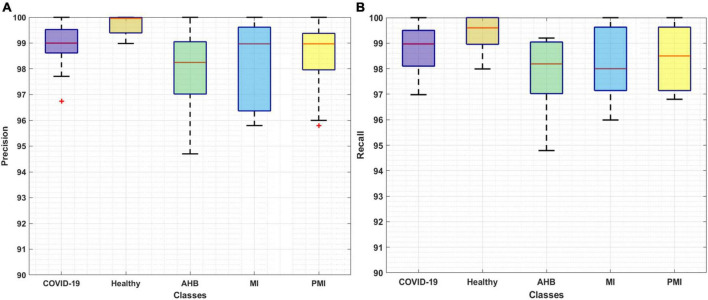
Evaluation of proposed method through **(A)** Precision and **(B)** Recall.

The classification results of the proposed Efficient-ECGNet for all five types of ECG images are calculated by using the precision, recall, accuracy, F1-measure, and error rate. Initially, we have plotted the box plots to show the obtained class-wise precision and recall values as these plots can better show the obtained results by demonstrating the lowest, highest, and average values along with the regularity and divergence of the data (Figure 6). Figure 6 is clearly showing that the proposed model is capable of accurately identifying and classifying the COVID-19 ECG images from all other four classes of normal and cardiac patients.

**FIGURE 7:**
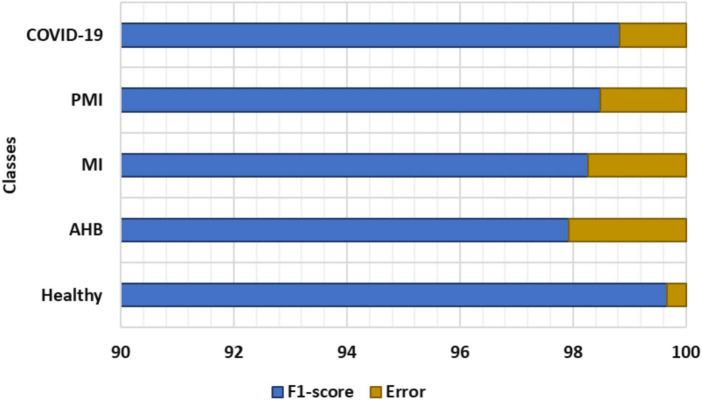
F1_score and Error rate of the proposed technique.

To further explain the recognition ability of the proposed solution for the COVID-19 virus from the ECG signal, we have drawn the F1-measure together with the error rates depicted in Figure 7. In Figure 7, the obtained F1-measure value is shown by using the blue color, while the rest area is presenting the values of error rates. It is quite visible from Figure 7 that our proposed solution is robust to classify all five types of images from the employed dataset by showing an average error rate of 1.3%. More explicitly, our Efficient-ECGNet has acquired the F1-score of 99.67, 97.92, 98.27, 98.49, and 98.83% for the normal, AHB, MI, PMI, and COVID-19-effected patients, respectively along with the minimum and maximum error rates of 0.33 and 2.08%. Moreover, the Efficient-ECGNet model shows enhanced class-wise classification accuracy values of 99.89, 97.72, 98.44, 98.46, and 98.81% for the normal, AHB, MI, PMI, and COVID-19-effected patients, respectively. The reported scores are clearly showing that our work is robust to differentiate the several types of ECG images and has an improved recall ability because of the reliable feature extraction power of our Efficient-ECGNet model.

**FIGURE 8:**
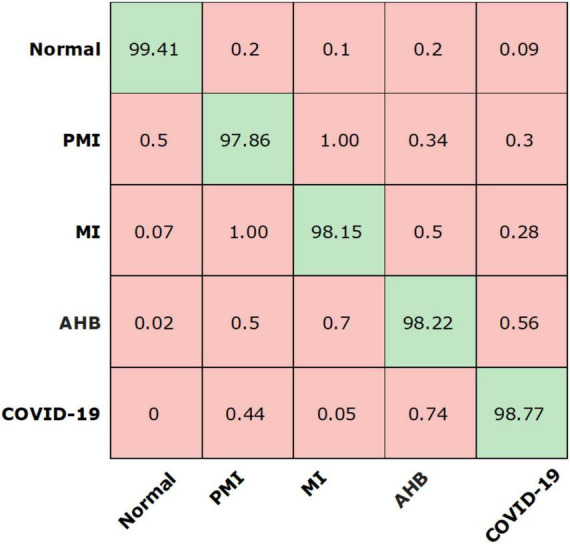
Confusion matrix of the presented work.

We have further explored the classification performance of the proposed model *via* plotting the confusion matrix as exhibited in Figure 8. This measure has the ability to better show the classification analysis of the introduced framework in terms of actual and estimated classes. More specifically, the Efficient-ECGNet has acquired the TPR of 99.41, 97.86, 98.15, 98.22, and 98.77 for the normal, AHB, MI, PMI, and COVID-19-effected patients, respectively. The confusion matrix reported in Figure 8 is clearly showing that there is little similarity found among the AHB and COVID-19 affected images, however, both are distinguishable.

**FIGURE 9:**
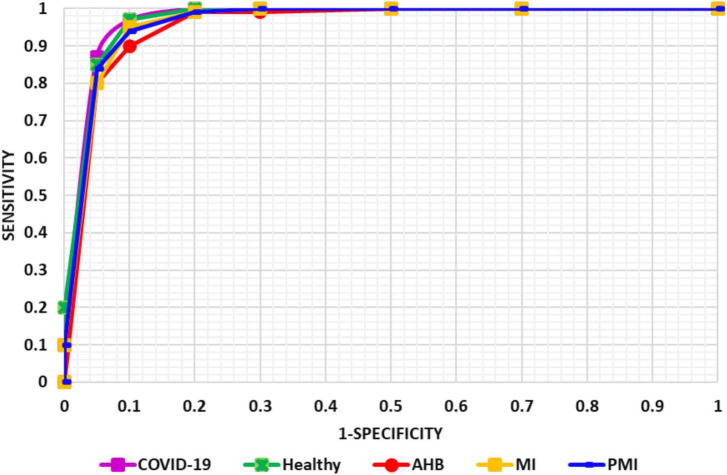
Evaluation through AUC measure.

To more explicitly discuss the classification results of the proposed solution, we have designed the AUC-ROC curves as given in Figure 9 for all five types of ECG samples. AUC-ROC plot is an essential performance analysis measure for validating the categorization ability of any model. In the AUC-ROC curve, the ROC shows the probability curve whereas the AUC area demonstrates the approximation of separability. In our work, the AUC-ROC curve is explaining the ability of the proposed solution to recall the samples of various classes, i.e., normal, MI, PMI, AHB, and COVID-19-effected patient ECG samples. In the AUC-ROC curve, as the value is moving toward 1, it exhibits the better performance of the framework and one can clearly see from Figure 9 that our model is proficient to differentiate the COVID-19 samples from all other types of ECG signals and thus have better recall rate.

The conducted experimentation is clearly showing that the introduced Efficient-ECGNet model is effective to detect and categorize the COVID-19 virus from the ECG signal and has the power to differentiate it from other normal and cardiac patient’s ECG images. The basic cause of the better performance of the Efficient-ECGNet model is because of the reliable image key-points computation ability which assists to show the complex ECG image transformations in a better way.

#### Explainability testing

**FIGURE 10:**
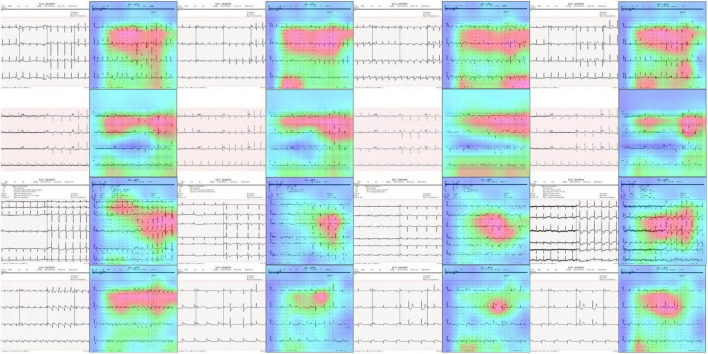
Heatmaps attained with the proposed approach.

One of the major requirements for an accurate disease classification model is that it must take the correct region of the suspected samples to accomplish the abnormality categorization task. To investigate this aspect, we performed an experiment to analyze the inner working of the proposed approach by obtaining the heatmaps as these are capable of determining whether the framework employs the precise pixels from the diseased region to produce the classification result. Such explainability of the model assists human experts to determine whether the output is trustworthy by analyzing whether the region of interest is existent in the heatmap areas. For this reason, we have used the Grad-CAM method (44) to obtain the heatmaps against the final convolution layer of the proposed approach. We employed the Grad-CAM method as it is a standard and more accurate method in comparison to other approaches like saliency maps to understand the black box nature of the DL frameworks. Grad-CAM has the ability to provide a better explanation of the model for distorted samples containing arbitrarily shaped affected areas. The attained results are shown in Figure 10. It can be seen from the results shown in Figure 10 that our approach is focusing on those areas where the abnormality exists which is showing the effective deep features computation of our model and assists it in better recognizing several ECG image-based diseases.

#### Similarity evaluation

**FIGURE 11:**
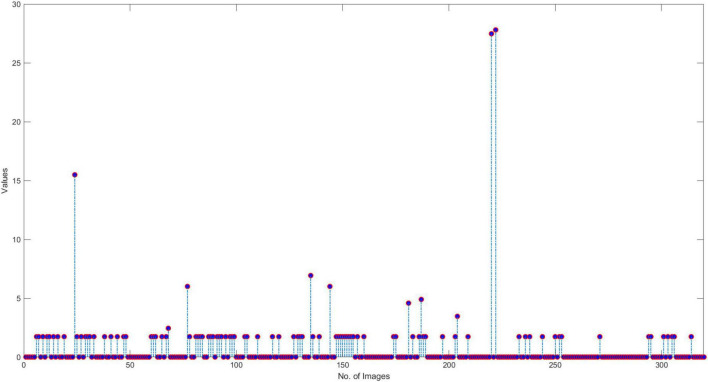
Similarity with Healthy class.

**FIGURE 12:**
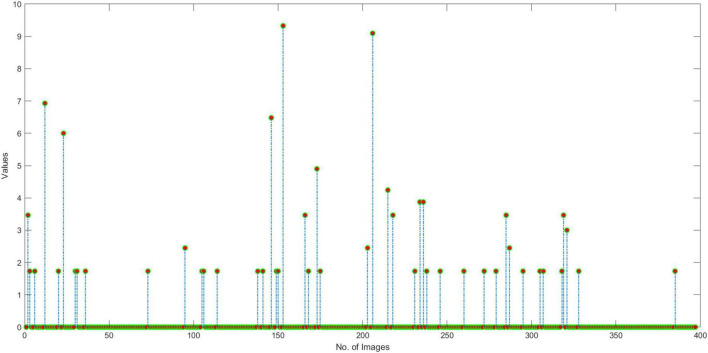
Similarity with AHB class.

**FIGURE 13:**
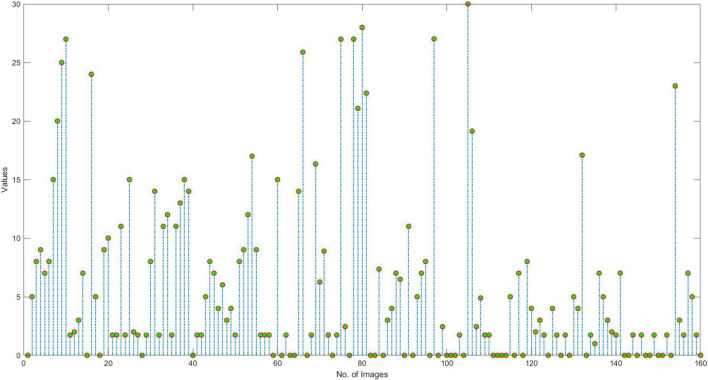
Similarity with MI class.

**FIGURE 14:**
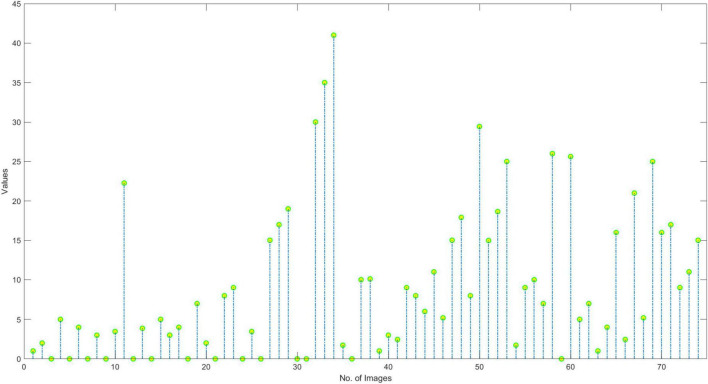
Similarity with PMI class.

In this section, we have elaborated on the similarity of COVID-19-affected ECG signal with other four classes namely normal, MI, PMI, and AHB, respectively. *It is important to highlight that to the best of our knowledge, it is the first work that has provided this type of analysis.* Initially, we have taken all the COVID-19 samples and then compute their distance from other classes to determine the level of resemblance. Few samples are shown in Figures 11–14 to show the visual demonstration of COVID-19 ECG images with the healthy, AHB, MI, and PMI-affected ECG images, respectively.

**FIGURE 15:**
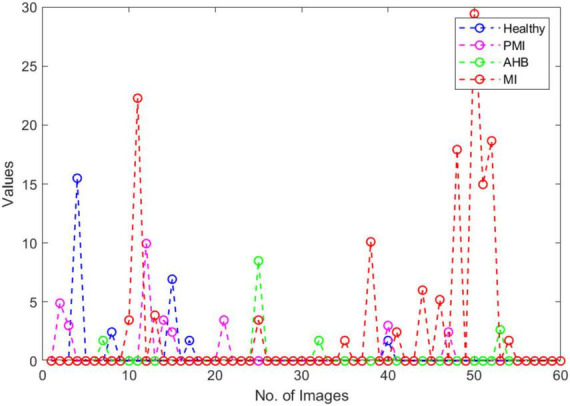
COVID similarity with other classes.

Furthermore, we have provided an overall representation of obtained results in Figure 15. From the results reported in Figures 11–14, it is quite evident that the COVID-19-affected ECG image has more resemblance to the AHB images. Although, a reasonable resemblance has been found with the MI class as well, however, the similarity with the AHB class is quite prominent. This is expected as the AHB being a respiratory disease should be more similar to COVID-19. *The resemblance of COVID and MI class shows a new discovery where we can conclude that there exists some sort of correlation between COVID and cardio disease, and patients catching up with COVID might be more vulnerable to developing any cardio disease.* Moreover, it is important to mention that the COVID-19-affected ECG images have the least similarity with the PMI class. So, based on this analysis, we can say that if a person is having an abnormal heartbeat then he can be a victim of COVID-19.

### Comparative analysis

Here, extensive experimentation is conducted to compare the coronavirus recognition ability of the introduced framework with the other contemporary methods. For this reason, we have used three types of investigation. Firstly, we evaluated the classification results of the proposed solution with the base approaches, then we evaluated it against other well-known DL frameworks. Finally, we have experimented to compare the results of our approach with several latest techniques to demonstrate the efficiency of the proposed work. A detailed demonstration of each experiment is given in the subsequent sections.

#### Comparison with base networks

In this experiment, the robustness of the proposed work is evaluated against several base methods namely EfficientNetV2-B0 (39), EfficientNetV2-B1 (39), EfficientNetV2-B2 (39), EfficientNetV2-B3 (39), and EfficientNetV2-B4 (39). To perform a fair comparison, we have designed two types of experiments.

Initially, the class-wise performance of the proposed approach is accomplished against the other base models, and the performance in the form of categorization accuracy is elaborated in Table 3. It can be seen from Table 3 that our approach exhibits higher classification accuracy for all five classes from the employed dataset namely Normal, AHB, MI, PMI, and COVID-19. In a more descriptive manner, the comparative base approaches show an average classification accuracy of 98.96% for the Normal class, which is 99.89% for our approach. Hence for the Normal class, the presented approach attains the performance gain of 0.93%. For the AHB class, the competitor methods obtained a 96.35% average accuracy score, comparatively, the proposed work gives an average accuracy value of 97.72%. So, for the AHB class, the presented methodology attains an average performance gain of 1.37%. Moreover, for the MI and PMI classes, the base techniques scored 97.57 and 97.72%, respectively, Whereas, the proposed Efficient-ECGNet approach shows the average accuracy values of 98.44 and 98.46% for the MI and PMI classes, respectively, and gives the average performance gain of 0.87 and 0.74%. Moreover, for the COVID-19 class, the proposed method scored 98.81%, which is 97.09% for the other base models. So, for the COVID-19 class, we have given an average performance gain of 1.72% which is showing the efficacy of the introduced framework.

**TABLE 3:** Class-wise performance in terms of accuracy with base models.

Model	Healthy	AHB	MI	PMI	COVID-19
EfficientNetV2-B0	98.33	95.88	97.21	97.29	96.77
EfficientNetV2-B1	98.12	95.81	97.23	97.31	96.28
EfficientNetV2-B2	99.17	96.59	97.58	97.46	96.48
EfficientNetV2-B3	99.53	96.61	97.72	98.16	97.69
EfficientNetV2-B4	99.66	96.84	98.13	98.36	98.21
**Efficient-ECGNet**	99.89	97.72	98.44	98.46	98.81

In the second experiment, we compared the performance of all peer approaches against our proposed method over the entire dataset. For this reason, we have chosen numerous standard measures named precision, recall, and accuracy, and obtained results are shown in Table 4. From Table 4, it is clearly visible that our work has attained the highest results in terms of all evaluation measures by showing the 98.74, 98.53, and 98.66% for the precision, recall, and accuracy metrics, respectively. The EfficientNetV2-B1 showed the lowest performance by exhibiting scores of 96.14, 96.44, and 96.95% for the precision, recall, and accuracy metrics, respectively. The second lower results are obtained by the EfficientNetV2-B0 having a score of 96.89, 96.31, and 97.10% for the precision, recall, and accuracy metrics, respectively. The EfficientNetV2-B4 exhibits comparable performance scoring 98.10, 97.88, and 98.24% for the precision, recall, and accuracy measures, respectively. More specifically, for the precision metric, the base methods exhibit an average value of 97.07%, which is 98.74% for our approach. So, the presented work gives an average performance gain of 1.67% for the precision metric. Similarly, for the recall and accuracy evaluation parameters, the base approaches show the average values of 96.86 and 97.54%, respectively. While in comparison, our approach shows the average values of 98.53 and 98.66% and gives the performance gain of 1.67 and 1.12%. So, we can say that in comparison to other base models, our approach is more proficient in the COVID-19 classification and can better recognize it from other normal and cardiac ECG images. The main distinction of our approach is the employment of fused MBConv blocks along with the added dense layers which assist it in better tackling the framework over-tuning problem. Such architecture of Efficient-ECGNet helps to better understand the complex structure of ECG images by feasibly presenting the stable sample key-points and giving it a performance improvement over its base models.

**TABLE 4:** Overall performance evaluation with base models.

Model	Precision (%)	Recall (%)	Accuracy (%)
EfficientNetV2-B0	96.89	96.31	97.10
EfficientNetV2-B1	96.14	96.44	96.95
EfficientNetV2-B2	97.02	96.62	97.46
EfficientNetV2-B3	97.18	97.04	97.94
EfficientNetV2-B4	98.10	97.88	98.24
**EfficientECG-Net**	98.74	98.53	98.66

#### Comparison with deep-learning-based methods

Here, we experimented to check the COVID-19 classification results of presented work against numerous contemporary DL-based methods namely MobileNetV2 (38), Resnet18 (45), Resnet50 (46), Resnet101 (47), Densenet201 (48), and InceptionV3 (49) as described in Rahman et al. (4). The comparative analysis in terms of precision, recall, accuracy, and F1-score is demonstrated in Table 5. One can see from the comparison given in Table 5 that the introduced framework has outperformed the other DL-based approaches in terms of all evaluation metrics. The minimum value is obtained by the ResNet-18 network with the precision, recall, accuracy, and F1-score of 95.44, 95.34, 95.34, and 95.28%, respectively. The second-lowest result is obtained by the MobilenetV2 which scored 96.29, 96.22, 96.22, and 96.2% for the precision, recall, accuracy, and F1-score, respectively. Comparatively, the proposed Efficient-ECGNet framework attains the highest results with the precision, recall, accuracy, and F1-score of 98.74, 98.53, 98.66, and 98.63%, respectively. More explicitly, for the precision measurement, the selected DL-based methods attain an average value of 96.71%, which is 98.74% for our approach. So, our method acquires a 2.03% performance gain. Similarly, for the recall evaluation parameter, the other DL-based techniques scored 96.67%, which is 98.53% for our case. Therefore, we have attained an average gain of 1.86%. Moreover, for the accuracy and F1-score, the comparative DL-based approaches show average values of 96.67 and 96.64%. Comparatively, our technique shows the average accuracy and F1-score of 98.66 and 98.63% and shows average gains of 1.99 and 1.99%, respectively. It can be said from the conducted analysis that the proposed solution is more effective in coronavirus-affected samples detection and categorization. The basic cause for the robust classification results of the presented method is the inclusion of the fused MBConv blocks which enables the Efficient-ECGNet approach to better nominates the reliable set of sample key-points. Moreover, the introduction of dense layers in the proposed architecture further enables it to better learn the complex transformations of ECG images which ultimately causes to enhance the recognition ability of the proposed solution.

**TABLE 5:** Performance comparison with DL-based approaches.

Model	Precision (%)	Recall (%)	Accuracy (%)	F1-score (%)
MobileNetV2	96.29	96.22	96.22	96.2
Resnet18	95.44	95.34	95.34	95.28
Resnet50	96.43	96.43	96.43	96.4
Resnet101	97.07	97	97	96.95
Densenet201	97.2	97.21	97.2	97.2
InceptionV3	97.82	97.83	97.83	97.82
**Proposed**	98.74	98.53	98.66	98.63

#### State-of-the-art comparison

Here, we have compared the ECG images-based COVID-19 classification performance of the proposed approach with the other latest methods from the history performing a similar task. To conduct an unbiased comparative examination, the average evaluation performance values of the approaches described in Rahman et al. (4) and Anwar and Zakir (7) are taken and compared with the average results of the proposed solution. The obtained comparison in the form of several standard metrics is shown in Table 6.

**TABLE 6:** Performance analysis of our approach with other latest methods.

Model	Precision (%)	Recall (%)	Accuracy (%)	F1-score (%)
Rahman et al. (4)	97.82	97.83	97.83	97.82
Anwar and Zakir (7)	80.8	75.80	81.80	77.40
**Proposed**	98.74	98.53	98.66	98.63

Rahman et al. (4) proposed an approach to locate and categorize the coronavirus-affected images from the ECG images *via* employing several DL-based approaches and obtained the best results for the InceptionV3 model with the values of 97.82, 97.83, 97.83, 97.82%, for the precision, recall, accuracy, and F1-score, respectively. Similarly, Anwar and Zakir (7) also utilized a DL-based approach to recognize and classify the COVID-19 samples from the ECG images and acquired an average precision, recall, accuracy, and F1-Score of 80.80, 75.80, 81.80, and 77.40%, respectively.

The performance evaluations shown in Table 6 is clearly explaining that our proposed approach is more reliable in coronavirus-affected samples detection and categorization as compared to the other latest methods. The presented approach achieved the average precision, recall, accuracy, and F1-score of 98.74, 98.53, 98.66, and 98.63%, respectively, which are greater than all the competitor methods. More clearly, for the precision measurement, the selected methods exhibit an average value of 89.31% which is 98.74% for the presented framework. Therefore, for the precision metric, our method provides a performance gain of 9.43%. Whereas for the recall evaluation parameter, the comparison methods obtain an average value of 86.82%, whereas the proposed framework acquires an average recall of 98.53%. Hence for the recall measurement, our approach gives a performance gain of 11.17%. Similarly, for accuracy, the peer approaches demonstrate an average value of 89.82%, whereas comparatively, the proposed solution attains the accuracy value of 98.66%. Therefore, for the accuracy evaluation performance measure, the introduced approach shows an average gain of 8.74%. Moreover, for the F1-score, the comparative methods give an average value of 87.61%, which is 98.63% for the presented work. Hence, we give an average performance gain of 11.02% for the F1-score measure. The conducted analysis is clearly depicting that the proposed Efficient-ECGNet model is more reliable and effective for COVID-19 classification from the ECG images as compared to the approaches mentioned in Rahman et al. (4) and Anwar and Zakir (7). The comparative techniques (4, 7) are using very large and complex network architecture, which ultimately causes the model over-tuning issue and enhances their computational processing complexity as well. Whereas, our work utilizes the fused MBConv blocks which minimizes the framework architectural complexity and reduced the computational burden as well. Moreover, the employment of dense layers permits the framework to extract a more discriminative feature vector which causes to enhance the recall ability of the proposed solution. Consequently, it can be said that the proposed work gives both a lightweight and effective solution to coronavirus-affected samples identification and categorization performance from the existing latest methods.

## Conclusion

In the presented approach, a DL framework has been presented to automatically identify and classify the coronavirus-affected samples by using the ECG-based image modalities. More descriptively, we have presented a customize EfficientNetV2-B4 model named Efficient-ECGNet with additional introduced dense layers to compute a more reliable set of image features and classify the suspected samples into five respective classes named normal, COVID-19, MI, PMI, and AHB. Furthermore, we have presented a module to measure the similarity of COVID-19-based ECG image with other classes and have found that coronavirus-based ECG image is more related to AHB disease. A detailed evaluation has been performed over a standard ECG images-based dataset to show the efficacy of our approach. Both the visual and numeric evaluations show that the presented framework is more effective for COVID-19-affected image recognition. Moreover, the inclusion of similarity estimation of COVID-19 samples against other classes can further assist doctors in better understanding the nature of this lethal virus. Further, we also provided an Explainability analysis to present the effective key-points computation ability of our model. Hence, it can be concluded that our approach can assist doctors in better curing the COVID-19 victims. The results of the proposed method can contribute to future research in both ML and DL areas. Even though we have attained better results, the presented technique requires to be evaluated on a large-scale dataset, so that it can be applied to other medical-related problems. In the future, we plan to test the proposed approach on COVID-19 variants-based ECG data based on the availability of such datasets. Moreover, we also plan to test the presented work on a portable ECG sensor-based dataset to further increase the significance of the system.

## Data availability statement

Publicly available datasets were analyzed in this study. This data can be found here: https://data.mendeley.com/datasets/gwbz3fsgp8/1.

## Ethics statement

Ethical review and approval was not required for the study on human participants in accordance with the local legislation and institutional requirements. Written informed consent from the patients/participants OR patients/participants legal guardian/next of kin was not required to participate in this study in accordance with the national legislation and the institutional requirements.

## Author contributions

AJ and TN: conceptualization. MN, TN, AJ, KM, and AS: methodology and writing—original draft preparation. MN and TN: software, data curation, and visualization. MK, MH, AT, and MA: validation and writing—review and editing. AJ and KM: formal analysis. AS, MK, and MH: investigation. AT and MA: resources. KM and AS: supervision. AJ, KM, AS, MK, and MH: project administration. AS: funding acquisition. All authors contributed to the article and approved the submitted version.
